# The Dangers of Cantharides

**Published:** 1875-09

**Authors:** 


THE DANGERS OF CANTHARIDES.—We see it stated in the dailies that two young men of Warsaw, Indiana, gave a young woman a dose of wine and cantharides, for an infamous purpose, and caused her death. They acknowledged the act, and are in jail.

Physicians should take proper occasions to disabuse the public mind of the belief that cantharides is an aphrodisiac, and also to warn of its dangerous character. Vile publications have disseminated a belief that it is a true venereal excitant, which it is not, in any sense.—*Med. and Surg. Reporter.*

